# Effects of Arm-Crank Exercise on Fitness and Health in Adults With Chronic Spinal Cord Injury: A Systematic Review

**DOI:** 10.3389/fphys.2022.831372

**Published:** 2022-03-17

**Authors:** Shin Yi Chiou, Emma Clarke, Chi Lam, Tom Harvey, Tom E. Nightingale

**Affiliations:** ^1^School of Sport, Exercise and Rehabilitation Sciences, University of Birmingham, Birmingham, United Kingdom; ^2^Centre for Human Brain Health, University of Birmingham, Birmingham, United Kingdom; ^3^MRC Versus Arthritis Centre for Musculoskeletal Ageing Research, University of Birmingham, Birmingham, United Kingdom; ^4^Centre for Trauma Sciences Research, University of Birmingham, Birmingham, United Kingdom; ^5^International Collaboration on Repair Discoveries, Faculty of Medicine, University of British Columbia, Vancouver, BC, Canada

**Keywords:** paraplegia, tetraplegia, balance, mobility, metabolic syndrome, upper-body exercise

## Abstract

Individuals with spinal cord injury (SCI) may benefit less from exercise training due to consequences of their injury, leading to lower cardiorespiratory fitness and higher risks of developing cardiovascular diseases. Arm-crank exercise (ACE) is the most common form of volitional aerobic exercise used by people with SCI outside a hospital. However, evidence regarding the specific effects of ACE alone on fitness and health in adults with SCI is currently lacking. Hence, this review aimed to determine the effects of ACE on cardiorespiratory fitness, body composition, cardiovascular disease (CVD) risk factors, motor function, health-related quality of life (QoL), and adverse events in adults with chronic SCI. Inclusion criteria were: inactive adults (≥18 years) with chronic SCI (>12 months post injury); used ACE alone as an intervention; measured at least one of the following outcomes; cardiorespiratory fitness, body composition, cardiovascular disease risk factors, motor function, health-related QoL, and adverse events. Evidence was synthesized and appraised using GRADE. Eighteen studies with a combined total of 235 participants having an injury between C4 to L3 were included. There was a moderate certainty of the body of evidence on ACE improving cardiorespiratory fitness. Exercise prescriptions from the included studies were 30–40 min of light to vigorous-intensity exercise, 3–5 times per week for 2–16 weeks. GRADE confidence ratings were very low for ACE improving body composition, CVD risks factors, motor function, or health-related QoL. No evidence suggests ACE increases the risk of developing shoulder pain or other injuries. Overall, this review recommends adults with chronic SCI should engage in regular ACE to improve cardiorespiratory fitness. More high-quality, larger-scale studies are needed to increase the level of evidence of ACE in improving cardiorespiratory fitness and to determine the effects of ACE on other outcomes.

**Systematic Review Registration:** [https://www.crd.york.ac.uk/prospero/display_reco rd.php?ID=CRD42021221952], identifier [CRD42021221952].

## Introduction

Spinal cord injuries (SCI) occur as a result of damage to the spinal cord, which has wide-ranging negative effects that depend on the severity of damage and level of lesion (World Health Organisation, 2013). Symptoms can range from partial to complete loss of sensation or muscular control over the trunk, legs, and arms as well as impairments of autonomic functions (e.g., cardiovascular control, temperature regulation, bladder and bowel control) and/or breathing (Jensen et al., 2007; Nas et al., 2015). As described in detail elsewhere (Cowley, 2018; Gee et al., 2021), high-level SCI (at or above the sixth thoracic level) can disrupt supra-spinal sympathetic control of the heart and blood vessels, which is required to initiate and then maintain an appropriate cardiovascular response to aerobic exercise. The extent to which the physiological response to exercise is impaired following SCI is dependent on the severity and level of injury (West et al., 2012, 2015). Indeed, following an autonomic and motor-sensory complete, cervical SCI (i.e., greatest disruption to brainstem-spinal sympathetic pathways), peak heart rate, circulating catecholamines and venous return are reduced, brachial blood pressure is low and stroke volume is restricted (Krassioukov and West, 2014; Cruz and Blauwet, 2018). Furthermore, the dimished descending sympathetic drive following high level SCI may impact substrate mobilization (lipolysis) during exercise and adipose tissue metabolism (Smith and Yarar-Fisher, 2016; Cowley, 2018). The aformentioned exercise-related physiological dysfunctions caused by high-level SCI can impact exercise capacity and may influence the magnitude of a training effect with aerobic exercise interventions in this population.

Following SCI there are several personal (including physical deconditioning and secondary conditions) and environmental factors that are antecedents to physical inactivity (Rimmer et al., 2012). Research suggests that individuals with SCI are ∼40% less physically active than able-bodied counterparts (van den Berg-Emons et al., 2010). A relatively sedentary lifestyle reduces cardiorespiratory fitness as well as increases the risks for developing chronic diseases [i.e., cardiovascular disease (CVD) including metabolic syndrome and other related diseases] in individuals living with SCI (Duckworth et al., 1980; Spungen et al., 2003; Lavela et al., 2006). While physical inactivity is an important environmental factor linked with reduced cardiorespiratory fitness, it is worth noting that reduced cardiorespiratory fitness and impaired cerebrovascular function have been reported even in elite, highly trained athletes with cervical SCI (Bhambhani et al., 1994; Phillips et al., 2017), emphasizing the pathological concerns of impaired bulbospinal sympathetic control in this population. Indeed, a recent systematic review concluded that there was inconclusive evidence that aerobic exercise improved health and exercise performance in individuals with cervical SCI (Figoni et al., 2021). Nevertheless, regular exercise for adults with chronic SCI is recommended to improve cardiorespiratory fitness and cardiometabolic health (van der Scheer et al., 2017; Farrow et al., 2020). However, this recommendation is based on evidence from various modalities of exercise, some of which are resource intensive to implement (i.e., require expensive equipment and trained personnel) and are thus less likely to be translated into community gym facilities or home-based exercise. Given maintaining physical fitness and health are long-term goals for people living with SCI, it is important to identify the efficacy of exercise modalities that can be simply administered outside of a rehabilitation setting, are affordable, safe and can be performed with minimum supervision.

Arm-crank exercise (ACE) has been shown to improve cardiorespiratory fitness and health in people with SCI (DiCarlo, 1988; Nightingale et al., 2018; Farkas et al., 2021). Studies have also shown that ACE in people with SCI improves wheelchair mobility (DiCarlo, 1988; Bresnahan et al., 2019) and health-related quality of life (QoL) (Nightingale et al., 2018). Furthermore, a recent study reported arm-crank ‘spin’ exercise classes improved seated balance with eyes closed (Williams et al., 2020). ACE uses ergometers that are often found in fitness centers/gyms, which are simple to set up relative to other types of exercise modalities. For example, setting up a FES leg-cycle ergometer is often intricate and time consuming (i.e., placing electrodes). ACE has the potential to be easily introduced into a home environment or in the local community, thereby overcoming environmental and psychosocial barriers to engage in physical activity reported in this population (Kehn and Kroll, 2009). Although hand-cycling shares similar advantages to ACE, a hand-cycling bike takes more space and requires individuals transferring from a wheelchair to the bike.

Other systematic reviews looking at the impact of exercise in this population have combined upper-limb exercise modalities (e.g., wheelchair propulsion, handcycling or arm-crank exercise) or combined with hybrid or lower-limb exercise strategies (van der Scheer et al., 2017; Farrow et al., 2020). Indeed, the current SCI-specific exercise guidelines are based on data from a mixture of upper and lower-body exercises (Martin Ginis et al., 2018). Absolute oxygen uptake is less in relative-intensity matched upper-body exercise compared to lower-body or whole-body exercise (Phillips et al., 1998; Calbet et al., 2005). Acute hybrid exercise (paired FES-evoked lower-limb cycling and handcycling) in individuals with SCI has demonstrated greater anti-inflammatory potential, higher metabolic demand and cardiorespiratory responses than handcycling alone (Bakkum et al., 2014; Paulson et al., 2014), implying that whole-body exercise might be more effective for improving body composition and cardiorespiratory fitness in this population. Therefore, relying on the pooled effects collated from multiple exercise modalities may overestimate the real effects from ACE alone as a monotherapy. Hence, there is a need to understand the specific effects of ACE in people living with SCI.

To the best of our knowledge, there are no systematic reviews that specifically synthesizes the effects of ACE for inactive adults with chronic SCI. The objectives of this review were to (a) identify the effects of ACE in individuals with chronic SCI; and (b) to determine a specific prescription for ACE to achieve improvements in cardiorespiratory fitness, body composition, CVD risk factors, motor function, and health-related QoL in individuals with chronic SCI.

## Materials and Methods

This review was pre-registered on PROSPERO (CRD42021221952) and conducted and reported according to PRISMA guidance (Page et al., 2021).

### Eligibility Criteria

Studies included adults (≥18 years) with traumatic and non-traumatic chronic SCI (>12 months post injury) who did not regularly participate in sports to ensure that the review can be generalized to the wider SCI population. All included studies tested an ACE intervention with no specific length of intervention required, however, studies with one-off sessions were excluded. Studies testing ACE combined with multiple forms of exercise (e.g., circuit-based resistance training interventions) were also excluded. This review included randomized controlled trials (RCTs), non-RCTs, and observational studies to optimize the searches used and provide an adequate number of papers. Case reports and cross-sectional studies were excluded due to their high potential for bias. The studies compared the effects of ACE using pre- and post-intervention values for specific outcome measures. In RCTs and non-randomized controlled trials, the comparisons were made with the control group, as well as pre-and post-training values. All included studies reported any of the following outcomes: cardiorespiratory fitness, risk factors associated with cardiometabolic syndrome (i.e., insulin sensitivity, lipid profiles, and markers of inflammation), body composition, motor function (i.e., muscle strength, balance, and community mobility), health-related QoL, and adverse events.

### Search Strategies and Study Selection

All searches were conducted by three authors (CL, EC, TH). Electronic database of PubMed, EMBASE, CINAHL, and Zetoc were searched on 12/03/2021. Searches were not restricted by date or design, however, studies not written in English were excluded. Hand-searching of the reference lists of all included papers and previous systematic reviews was also carried out; an additional search on PubMed was carried out in September 2021 to identify any new publications between March and September 2021.

In order to increase the responsiveness of the search, the [MeSH] function was used for ‘*spinal cord injuries,’ ‘paraplegia,’* and *‘tetraplegia’* (or ‘*quadriplegia*’ if preferred term of the database). The Boolean terms ‘AND’ and ‘OR’ were used to combine two search strings together with the [MeSH] term followed by the exercise intervention terms; ‘*arm ergometer,’ ‘arm cycling,’ ‘arm crank,’ ‘arm exercise,’* and *‘arm training.’* A pilot search of electronic databases was carried out prior to the main search to clarify the key words used in the search strategy while prioritizing a focus on the sensitivity of the search terms. The full search strategy for PubMed is presented in Supplementary Table 1.

Three of the authors (CL, EC, TH) conducted study selections. After duplicates were removed from the initial search, the title and abstract were screened independently by two of these three authors. Studies that remained after the screening process, or where eligibility was unknown, were read independently in full by the three authors. If there was inconsistency between the authors, the fourth author (SC) made the final decision.

### Data Collection and Management

After eligible studies were identified, a data extraction form was constructed using the Cochrane data extraction template, incorporating inclusion criteria and objectives for this review (Li et al., 2021). This was piloted prior to use and altered as needed. Data extraction was conducted by two of the three authors (TH, EC, CL); any disagreements or conflicts were discussed and assessed by a fourth author. The items extracted included: paper details (title, date, and authors), details of the participants (sample size, age, inclusion/exclusion criteria, level and severity of SCI, years since injury), intervention parameters (frequency, duration, and intensity), participant adherence (as stated in text), outcome measures used (primary and secondary outcome parameters), pre- and post-intervention outcome values, and adverse events.

### Quality Assessment

Risk of bias (RoB) for each study was evaluated independently by all authors based on the study design, data management, and reporting bias using the revised Cochrane tools: the RoB2 Cochrane’s tool for RCTs, the ROBINS-I tool for non-RCTs (Sterne et al., 2016, 2019), and the National Institutes of Health (NIH) quality assessment tool for before-after (Pre-Post) studies with no control group. In the event of any disagreements in RoB evaluation, group discussion took place to reach an overall consensus. The certainty of the body of evidence was assessed using the Grading of Recommendations Assessment, Development, and Evaluation (GRADE) framework (Guyatt et al., 2008).

### Analysis

Outcomes were grouped into five categories: (1) cardiorespiratory fitness, (2) body composition, (3) CVD risk factors, (4) motor function, and (5) health-related QoL for synthesis and grading the body of evidence. This was to reduce variability of outcome measures used between studies. Descriptive information (i.e., study design, demographic characteristics of participants), outcomes of the studies, and exercise prescriptions from the extracted data were summarized in tables to facilitate narrative synthesis for each outcome. Mean ± Standard deviation changes of all outcome measurements were extracted for calculating *Hedges’ g* effect sizes (Lakens, 2013).

## Results

**FIGURE 1:**
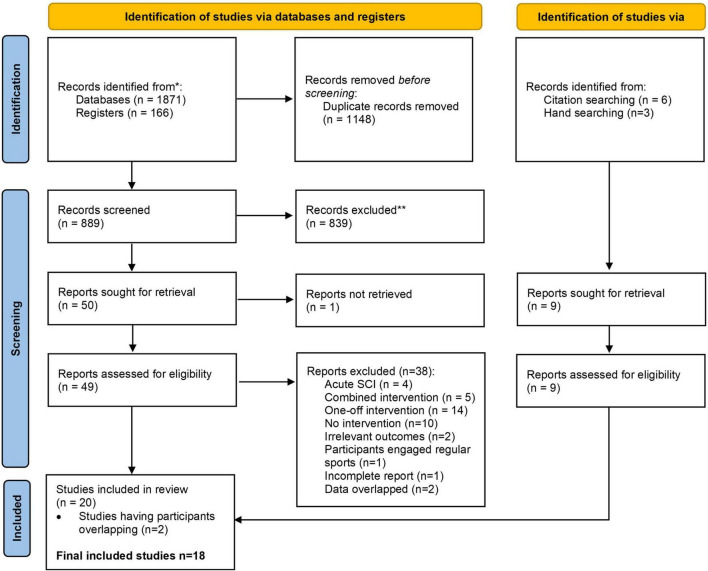
PRISMA flow diagram.

We identified 889 references, of which 839 were excluded following title and abstract screening. An additional nine articles were identified from hand-searching of relevant systematic reviews. Finally, 20 articles were included in the review (Figure 1).

### Characteristics of Included Studies

Study characteristics and the outcomes are summarized in Tables 1, 2. Included studies were published between 1988 and 2021. Ordonez et al. (2013) and Rosety-Rodriguez et al. (2014), as well as Nightingale et al. (2017b,2018) reported different outcome measures from the same group of participants; the results from these corresponding studies by the same authors were combined and reported as a single study. Since the purpose of this review was to determine the effects of ACE in chronic SCI, studies comparing ACE with a different type of intervention [e.g., whole-body exercise (Alrashidi et al., 2021), strength training (Jacobs, 2009), functional electrical stimulation (Farkas et al., 2021)] or comparing SCI with non-injured adults (El-Sayed and Younesian, 2005; Horiuchi and Okita, 2017) were treated as pre-post studies without a control group and only data from the ACE group were extracted. Three RCTs, one non-RCT, and thirteen pre-post studies were identified.

**TABLE 1:** Detailed findings from specific studies with a control group in the systematic review.

Author Design, RoB	Number, AIS, LOI	Age (years) Mean ± SD	Outcomes	Baseline, INT (CON) Mean ± SD	Δ Change, INT (CON)	*P*-value	Hedges’ *g*^
Davis et al., 1987 Non-RCT, Serious^$^	14 Paraplegia	30 ± 3	**V̇O2peak (L/min)**	**1.48 ± 0.10** **(1.55 ± 0.13)**	**0.46 (–0.07)**	**<0.05**	**3.54**
			
			SBP (kPa)	16.3 ± 0.7 (15.2 ± 0.8)	0.5 (2.4)	NS	–
			
			DBP (kPa)	10.4 ± 0.7 (10.8 ± 0.5)	–0.3 (0.8)	NS	–

Davis et al., 1991 RCT, High#	22 NI	30.1 ± 3.6	**V̇O2peak (L/min)**	**1.42 ± 0.3** **(1.66 ± 0.15)**	**0.22 (0.05)**	**<0.05**	**0.93**

Dyson-Hudson et al., 2007 RCT, High#	23 B, C5-L2	40.9 ± 8.8	Wheelchair User’s Shoulder Pain Index	11.5 ± 17.3 (8.4 ± 9.5)	–3 (2.8)	NS	–

Ordonez et al., 2013; Rosety-Rodriguez et al., 2014 RCT, Low#	17 A, ≥T5	29.6 ± 3.6	**V̇O2peak (mL/kg/min)**	**23.2 ± 2.1** **(23.0 ± 2.2)**	**2.4 (–)**	**0.031** *	**–**
			
			Body mass index (kg/m2)	27.6 ± 4.1 (27.8 ± 4.4)	–0.2 (–)	NS	–
			
			**Waist circumference (cm)**	**98.1 ± 6.6** **(98.4 ± 6.7)**	** –3.7 (–)**	**0.046**	**–**
			
			**Leptin (ng/mL)**	**9.6 ± 2.7** **(9.8 ± 2.8)**	** –2.1 (0.1)**	**<0.05**	**0.94**
			
			Adiponectin	18.8 ± 4.1 (18.5 ± 4.2)	0.6 (0.1)	NS	–
			
			PAI-1 (ng/mL)	29.8 ± 6.2 (30.2 ± 6.1)	–0.7 (–0.1)	NS	–
			
			**Tumor necrosis factor-α (pg/mL)**	**23.3 ± 5.6** **(23.6 ± 5.5)**	** –2.7 (–0.1)**	**<0.05**	**0.54**
			
			**Interleukin-6 (pg/mL)**	**6.7 ± 2.2** **(6.9 ± 2.3)**	** –2.6 (0.1)**	**<0.05**	**1.35**

Nightingale et al., 2017b,^2018^ RCT, Low^#^	21 A-D, T4-L3	47 ± 8	**V̇O2peak (mL/kg/min)**	**18.3 ± 4.9** **(18.8 ± 6.2)**	**3.4 (–0.5)**	**<0.05**	**0.67**
			
			**Peak Power (W)**	** – (–)**	**19 (–)**	**<0.05**	** –**
			
			**Body mass (kg)**	**76.8 ± 13.3** **(76.8 ± 1.3)**	** –1.1 (–0.7)**	**<0.05**	**0.03**
			
			Fat mass (kg)	27.6 ± 10 (25.5 ± 6.6)	–0.6 (0)	NS	–
			
			Lean body mass (kg)	45.6 ± 7.5 (47.7 ± 11)	–0.3 (–0.5)	NS	–
			
			SBP (mmHg)	128 ± 23 (128 ± 15)	–3 (–2)	NS	–
			
			DBP (mmHg)	77 ± 15 (81 ± 13)	–1 (–4)	NS	–
			
			**Fasting insulin (pmol/L)**	**54.8 ± 30.1** **(41.3 ± 18)**	** –12.7 (3.1)**	**<0.05**	**0.55**
			
			HDL-C (mmol/L)	1.1 ± 0.3 (1.0 ± 0.2)	0.1 (0.0)	NS	–
			
			LDL-C (mmol/L)	3.2 ± 0.9 (3.5 ± 0.8)	0 (–0.2)	NS	–
			
			Fasting glucose (mmol/L)	5.3 ± 0.5 (5.7 ± 1.3)	0.0 (0.0)	NS	–
			
			Non-esterified fatty acids (mmol/L)	0.6 ± 0.3 (0.7 ± 0.6)	0.3 (–0.1)	NS	–
			
			**HOMA-IR**	**1.03 ± 0.6 (0.8 ± 0.3)**	**–0.24 (0.06)**	**<0.05**	**0.53**
			
			**SF36 physical component**	**55 ± 20 (66 ± 9)**	**15 (1)**	**0.017**	**0.75**
			
			SF36 mental component	68 ± 23 (81 ± 12)	13 (–1)	NS	–
			
			Wheelchair User’s Shoulder Pain Index	13 ± 11 (19 ± 21)	0 (–5)	NS	–

*SD, standard deviation; RCT, randomized controlled trial; CON, control group; INT, intervention group; NS, non-significant; RoB, risk of bias; AIS, American Spinal Injury Association Impairment Scale; LOI, level of injury; V̇O_2peak_, peak oxygen consumption; HDL-C, high-density lipoprotein cholesterol; LDL-C, low-density lipoprotein cholesterol; PAI-1, plasminogen activator inhibitor 1; HOMA-IR, homeostasis model assessment of insulin resistance; SBP, systolic blood pressure; DBP, diastolic blood pressure; SF36, short-form 36.*

*Numbers in **bold** indicate a statistically significant change after the ACE.*

*– Not provided, unable to calculate, or not applicable; NI, no information.*

*^ Between-group effect sizes.*

*Comparison between pre- and post-training in INT.

*^#^Risk of bias assessed using Cochrane risk-of-bias tool for randomized trials (RoB2).*

*^$^Risk of bias assessed using The Risk Of Bias In Non-randomized Studies – of Interventions (ROBINS-I) assessment tool.*

**TABLE 2:** Detailed findings from specific pre–post design studies in the systematic review.

Author Design, Quality*	Number, AIS, LOI	Age (years) Mean ± SD	Outcomes	Baseline, Mean ± SD	Δ Change	*P*-value	Hedges’ *g*
DiCarlo, 1988 Pre-post, Fair	8 NI, C5-7	24 ± 4.0	**V̇O2peak (mL/kg/min)**	**12.1 ± 0.5**	**11.4**	**<0.05**	**5.14**
			
			**Mean Physical workload capacity (kg⋅m/min)**	**206 ± 6.8**	**49**	**<0.05**	**5.91**
			
			**Wheelchair propulsion (m)**	**1180 ± 100**	**920**	**<0.05**	**7.59**

McLean and Skinner, 1995 Pre-post, Fair	14 NI, C5-T1	33.8 ± 9.6	**V̇O2peak (mL/min)**	**720**	**60**	**<0.01**	**–**
			
			**Peak Power (W)**	**29.3**	**4**	**<0.05**	**–**

Silva et al., 1998 Pre-post, Fair	12 A, T1-L2	Median (range): 31 (22-54)	**FVC (L)**	**4.05**	**0.35**	**<0.01**	**–**
			
			MVV (L/min)	175	3	NS	–

El-Sayed and Younesian, 2005 Pre-post, Poor	5 NI, below T10	31 ± 2.9	**V̇O2peak (mL/kg/min)**	**24.1 ± 2.6**	**2.1**	**<0.05**	**0.94**
			
			**Peak workload (W)**	**168 ± 38**	**17**	**<0.05**	**0.50**
			
			**HDL-C (mmol/L)**	** –**	**↑**	**<0.05**	** –**
			
			**Triglycerides (mmol/L)**	** –**	**↓**	**<0.05**	** –**

Jacobs, 2009 Pre-post, Poor	9 A-B, T6-10	29.0 ± 9.9	**V̇O2peak (L/min)**	**1.27 ± 0.54**	**0.15**	**<0.05**	**0.25**
			
			**Peak Power (W)** ^#^	**308.8 ± 137**	**7.1**	**<0.05**	**0.05**
			
			Overhead press (kg)	39.8 ± 17.1	–0.1	NS	–
			
			Chest press (kg)	26.8 ± 9.9	–1.1	NS	–
			
			Seated dips (kg)	50.1 ± 12.2	–0.9	NS	–

Harnish et al., 2017 Pre-post, Poor	8 A-D, T1-L2	50.5 ± 9.0	**V̇O2peak (L/min)**	**1.4 ± 0.4**	**0.12**	**<0.05**	**0.19**
			
			**Peak Power (W)**	**84.4 ± 25.3**	**13.1**	**<0.05**	**0.51**
			
			Non-esterified fatty acids (mEq/L)	**1.35 ± 0.4**	** –0.34**	**<0.05**	**0.79**
			
			Fasting insulin (mU/l)	168.5 ± 78.5	–29.5	NS	–
			
			Fasting glucose (mg/dL)	76.6 ± 10.4	–0.4	NS	–

Horiuchi and Okita, 2017 Pre-post, Poor	9 A-B, T8-L1	38 ± 10	**V̇O2peak (mL/kg/min)**	**28.9 ± 4.1**	**3.8**	**<0.05**	**0.90**
			
			**Body mass (kg)**	**61 ± 7.0**	** –1.9**	**<0.05**	**0.26**
			
			**Waist circumferences (cm)**	**85.5 ± 6.2**	** –1.9**	**<0.05**	**0.32**
			
			**SBP (mmHg)**	**136 ± 5**	** –3**	**<0.05**	**0.68**
			
			DBP (mmHg)	75 ± 8	–2	NS	–
			
			HDL-C (mg/dL)	56 ± 7	2	NS	–
			
			LDL-C (mg/dL)	114 ± 24	–4	NS	–
			
			**Triglycerides (mg/dL)**	**154 ± 69**	** –38**	**<0.05**	**0.60**
			
			**PAI-1 (ng/dL)**	**52 ± 11**	** –14**	**<0.05**	**1.22**
			
			Hemoglobin A1c (%)	4.9 ± 0.6	–0.1	NS	–
			
			Fasting glucose (mL/dL)	102 ± 25	–3	NS	–
			
			Handgrip strength (kg)	50.4 ± 5.5	1.8	NS	–

Bresnahan et al., 2019 Pre-post, Fair	10 A-B, C7-T5	36.7 ± 12.5	**V̇O2peak (ml/kg/min)**	**10.8 ± 3.6**	**2**	**0.027**	**0.74**
			
			**Power (W)**	**40 ± 16**	**14**	**0.026**	**1.20**
			
			**12-min wheelchair propulsion (m)**	**628.50 ± 355.70**	**102.41**	**0.028**	**0.39**
			
			Fat mass (kg)	25.1 ± 11.9	–0.3	0.75	–
			
			Lean body mass (kg)	44.31 ± 10.3	0.52	0.75	–
			
			**Fasting insulin (μU/ml)**	**12.23 ± 5.58**	** –4.58**	**0.028**	**1.05**
			
			HDL-C (mg/dL)	36.33 ± 6.31	–1.5	0.07	–
			
			LDL-C (mg/dL)	104.83 ± 14.93	12	0.12	–
			
			Triglycerides (mg/dL)	164.5 ± 132.1	–44.5	0.6	–
			
			Fasting glucose (mg/dL)	99.83 ± 14.8	–0.83	0.92	–
			
			**HOMA-IR**	**1.6 ± 0.7**	** –0.6**	**0.04**	**1.09**

Graham et al., 2019 Pre-post, Fair	7 A-D, C6-L1	50.3 ± 1.3	**V̇O2peak (ml/kg/min)**	**12.5 ± 4.1**	**2.1**	**0.048**	**0.5**
			
			Power (watts)	313 ± 101	30	NS	–
			
			Body mass (kg)	90.3 ± 23.9	–0.5	NS	–
			
			Fat mass (kg)	37.8 ± 17.3	–0.5	NS	–
			
			Lean body mass (kg)	49.5 ± 9.1	–0.8	NS	–
			
			SBP (mmHg)	121.8 ± 21.3	–4.9	NS	–
			
			DBP (mmHg)	69.7 ± 12.1	–0.95	NS	–
			
			Fasting insulin (mL/dL)	21.7 ± 17.7	–12.7	NS	–
			
			HDL-C (mg/dL)	52.8 ± 7.9	–0.85	NS	–
			
			LDL-C (mg/dL)	98.0 ± 37.5	–9.25	NS	–
			
			Triglycerides (mg/dL)	93.3 ± 42.1	3.3	NS	–
			
			Fasting glucose (mL/dL)	132.0 ± 75.8	–9.9	NS	–
			
			HOMA-IR	9.2 ± 10.8	–6.6	NS	–
			
			Overhead press (kg)	41.6 ± 14.1	2.35	NS	–
			
			Triceps extension (kg)	28.7 ± 12	6.5	NS	–
			
			**Chest press (kg)**	**65.7 ± 24.9**	**3.8**	**0.04**	**0.15**
			
			**Lat pulldown (kg)**	**41.2 ± 10.4**	**9.3**	**0.02**	**0.67**

Brizuela et al., 2020 Pre-post, Fair	11 A-B, C4-7	36.5 ± 10.0	FVC (L)	2.6 ± 0.7	0.2	NS	–
			
			**MVV (L/min)**	**82.9 ± 29.5**	**3.2**	**0.03**	**0.31**
			
			**Power (W/kg)**	**0.7 ± 0.3**	**0.2**	**<0.01**	**0.71**

Williams et al., 2020 Pre-post, Good	14 A-D, C4-T12	44.3 ± 10.4	**V̇O2peak (ml/kg/min)**	17.1 ± 4.9	**2.7**	**0.005**	**0.56**
			
			**Power (W)**	84.8 ± 39.0	**12**	**0.012**	**0.30**
			
			Static balance, eyes open	–	–	NS	–
			
			**Static balance, eyes close**	** –**	**↑**	**0.013**	** –**
			
			Dynamic balance (mm)	387.5 ± 176.3	20.9	NS	–

Alrashidi et al., 2021 Pre-post, Fair	14 A-B, C4-T6	42 ± 10	**V̇O_2peak_ (ml/kg/min)**	**12.5 ± 6.7**	**3.0**	**<0.05**	**0.55**
			
			Peak Power (W)	47 ± 30	14	NS	–
			
			HDL-C (mmol/L)	1.2 ± 0.3	0.1	NS	–
			
			LDL-C (mmol/L)	2.7 ± 0.9	0.1	NS	–
			
			Triglycerides (mmol/L)	1.2 ± 0.6	0.0	NS	–
			
			Fasting glucose (mmol/L)	4.7 ± 0.5	0.0	NS	–
			
			HOMA-IR	0.26 ± 0.03	–0.02	NS	–
			
			Hemoglobin A1c (%)	5.2 ± 0.3	0.0	NS	–

Farkas et al., 2021 Pre-post, Fair	7 A-B, T4-10	42 ± 11	**V̇O_2peak_ (mL/kg/min)**	**15.91 ± 2.61**	**3.56**	**0.003**	**0.99**
			
			**Peak Power (W)**	**12.13 ± 6.45**	**31.68**	**<0.001**	**4.07**
			
			Body mass (kg)	79.1 ± 12.2	–0.5	NS	–
			
			Body mass index (kg/m^2^)	27.8 ± 3.4	–0.2	NS	–
			
			Waist circumferences (cm)	91.1 ± 9.6	–1.5	NS	–
			
			**Fat mass (kg)**	**29.6 ± 6.4**	**–1.9**	**0.05**	**0.32**
			
			Lean mass (kg)	46.6 ± 8.7	1	NS	–
			
			**SBP (mmHg)**	**117.7 ± 16.6**	**–9.7**	**0.032**	**0.60**
			
			DBP (mmHg)	59.9 ± 8.3	7.5	NS	–
			
			**Fasting insulin (μm/mL)**	**8.7 ± 8.5**	**–2.9**	**0.009**	**0.37**
			
			HDL-C (mg/dL)	35.7 ± 6.4	–2.3	NS	–
			
			LDL-C (mg/dL)	118.3 ± 30.5	0	NS	–
			
			Triglycerides (mg/dL)	104.7 ± 50.6	–1.1	NS	–
			
			Fasting glucose (mg/dL)	98.7 ± 11.6	–5.9	NS	–

*SD, standard deviation; NS, non-significant; AIS, American Spinal Injury Association Impairment Scale; LOI, level of injury; V̇O_2peak_, peak oxygen consumption; HDL-C, high-density lipoprotein cholesterol; LDL-C, low-density lipoprotein cholesterol; HOMA-IR, homeostasis model assessment of insulin resistance; PAI-1, plasminogen activator inhibitor 1; SBP, systolic blood pressure; DBP, diastolic blood pressure; FVC, forced vital capacity; MVV, maximal voluntary ventilation.*

*Numbers in **bold** indicate a statistically significant change after the ACE.*

*– Not provided, unable to calculate, or not applicable; NI: no information.*

*Quality assessed using The National Institutes of Health (NIH) quality assessment tool for before-after (Pre-Post) study with no control group.

*^#^Upper extremity Wingate anaerobic power testing.*

Sample sizes of each study ranged from 5 to 23 participants. There were a total of 235 individuals with SCI from 18 included studies in this review. Mean age was 37 years (standard deviation: 8 years), individuals with both complete and incomplete SCI were included [American Spinal Injuries Impairment Scale A–D], and level of injury ranged from C4 to L3.

Exercise intensity classification and prescriptions are summarized in Tables 3, 4. Intervention duration ranged from 2 to 16 weeks, with a frequency of 2–5 times a week. Time spent performing ACE per exercise session ranged from 20 to 40 min per session, with the majority of studies including a warm-up and cool-down as part of the intervention. Exercise intensity was commonly prescribed based on a percentage of peak oxygen consumption (V̇O_2peak_) (5/17), peak heart rate (for level of injury below T6; 3/17) or peak heart rate reserve (for level of injury above T6; 3/17), or using ratings of perceived exertion (e.g., Borg CR10 scale or Borg RPE 6-20 scale; 3/17). In addition, exercise intensity in each study was defined as either light, moderate, moderate-to-vigorous, or vigorous-intensity based on the classification of the American College of Sport Medicine (Mitchell et al., 2019; American College of Sports, 2021) (Table 3); one study used light-intensity (Brizuela et al., 2020), three studies employed moderate-intensity (McLean and Skinner, 1995; Dyson-Hudson et al., 2007; Farkas et al., 2021), nine applied moderate-to-vigorous-intensity (Davis et al., 1987; DiCarlo, 1988; El-Sayed and Younesian, 2005; Ordonez et al., 2013; Horiuchi and Okita, 2017; Nightingale et al., 2017b; Graham et al., 2019; Williams et al., 2020; Alrashidi et al., 2021), and four trained at vigorous-intensity (Silva et al., 1998; Jacobs, 2009; Harnish et al., 2017; Bresnahan et al., 2019). Arm cranking speed was between 50 and 60 revolutions per minute. ACE was delivered as group exercise in one study (Williams et al., 2020) and as high-intensity interval training in two studies (Harnish et al., 2017; Graham et al., 2019).

**TABLE 3:** Classification of exercise intensity.

	%V̇O_2peak_	%HR_peak_	%HR_reserve_	Rating of Perceived Exertion (RPE)
Light	37–45	57–63	30–39	Borg CR 10 < 3 or RPE 9–11
Moderate	46–63	64–76	40–59	Borg CR 10 3–4 or RPE 12–13
Vigorous	64–90	77–95	60–89	Borg CR 10 5–7 or RPE 14–17

*Table adapted from Mitchell et al. (2019) and Garber et al. (2011).*

*V̇O_2peak_, peak oxygen consumption; HR_peak_, peak heart rate; HR_reserve_, heart rate reserve.*

**TABLE 4:** Prescriptions of arm-crank exercise (ACE) from the included studies.

Author	Type	Time per session (minutes)	Frequency (/week)	Time per week (main exercise)	Duration (week)	Total number of sessions	Intensity
**Light-intensity**							
Brizuela et al., 2020	Supervised ACE	15–40-min ACE, time increased progressively	2x	30–80-min	8	16	Borg CR 10 scale between 2 and 3
**Moderate-intensity**							
McLean and Skinner, 1995	Supervised ACE	3-min warm-up, 20-35-min ACE, 3-min cool-down	3x	60–105-min	10	30	Initially 60% of peak power; peak power increased by 1watt each time if participants completed ACE for 35-min for two sessions.
Dyson-Hudson et al., 2007	Supervised ACE	20-min ACE	3x	60-min	12	36	70% of HR_peak_ (below T6) or Borg RPE (6–20) at moderate (at T6 and above), 60RPM
* Farkas et al., 2021	Supervised ACE	10-min warm-up, 40-min ACE, 10-min cool-down	5x	200-min	16	80	75% of HR_peak_ at 50RPM
**Moderate-to-vigorous intensity**							
Davis et al., 1987	Supervised ACE	20–40-min ACE	3x	60–120-min	16	48	50 or 70% V̇O_2peak_
DiCarlo, 1988	Supervised ACE	15–35-min ACE	3x	45–105-min	8	24	50–60% of HR_reserve_ at 50–60 RPM
Davis et al., 1991	Supervised ACE	Short-duration: 20-min ACE; Long-duration: 40-min ACE	3x	60–120-min	8	24	High-intensity: 70% V̇O_2peak_ Low-intensity: 50% V̇O_2peak_
* El-Sayed and Younesian, 2005	Supervised ACE	5-min warm up, 30-min ACE	3x	90-min	12	36	60–65% V̇O_2peak_
Ordonez et al., 2013; *Rosety-Rodriguez et al., 2014	Supervised ACE	10–15-min warm up, 20–30-min ACE, 5–10-min cool-down	3x	60–90-min	12	36	50–65% of HR_reserve_, increasing 5% every 3 weeks; ACE time increased progressively
* Horiuchi and Okita, 2017	Supervised ACE	2 × 30-min ACE, with a 10-min resting interval	4x	240-min	10	40	50–70% of HR_reserve_
*Nightingale et al., 2017b,^2018^	Unsupervised ACE	30-45-min ACE, extended 5-min/week.	4x	120–180-min	6	24	60% V̇O_2peak_ first 3 weeks; 65% V̇O_2peak_ final 3 weeks.
Graham et al., 2019	Supervised ACE	MIT: 30-min HIIT: 20-min, consisting of 30s (high intensity) x 4 repeats, 4-min (low intensity)	MIT: 3x HIIT: 2x	40–90-min	6	12–18	MIT: 55–65% V̇O_2peak_ HIIT: 4-min at 25% of heart rate reserve, followed by 30 s at 50% of peak power.
Williams et al., 2020	ACE, supervised group “spin” class	10-min warm-up, 40-min ACE, 10-min cool-down	3x	120-min	5	15	Borg CR 10 scale between 5.8 and 7.5, target RPE changes during each interval (mixed)
Alrashidi et al., 2021	Supervised ACE	30-min ACE	3x	90-min	24	72	Borg RPE 11-16
**Vigorous-intensity**							
Silva et al., 1998	Supervised ACE	30-min ACE	3x	90-min	6	18	HR corresponding to participants ventilatory threshold
Jacobs, 2009	Supervised ACE	30-min ACE	3x	90-min	12	36	70–85% of HR_peak_
* Harnish et al., 2017	Supervised sprint ACE	10-min warm-up; 30-sec each sprint, followed by 5-min slow pedaling, 4–7 sprints; 2-min cool-down	∼3x	66–116-min	∼2	6	Sprint against a resistance of 3.5% body weight for 30 s; 4–7 sprints, increasing progressively.
* Bresnahan et al., 2019	Supervised ACE	5-min warm-up, 30-min ACE; 5-min cool-down	3x	90-min	10	30	70% V̇O_2peak_ (re-assessed at week 5) at 50RPM; ACE time increased progressively week-by-week

*Borg CR 10, Borg rating of perceived exertion (RPE) scale 1-10; RPM, revolutions per minute; MIT, moderate-intensity training; HIIT, high-intensity interval training; V̇O_2peak_, peak oxygen consumption; HR_peak_, peak heart rate; HR_reserve_, heart rate reserve.*

*Indicates improvement in cardiovascular results after the ACE.

RoB for studies with a control group is shown in Table 1 and in Supplementary Table 2. Reasons for studies being rated at moderate to high RoB were inadequate randomization process, missing outcome data, uncontrolled confounding factors (e.g., level of injury, severity of injury), subjective outcome measures (i.e., wheelchair user’s shoulder pain index, a self-reported instrument) that may likely be influenced by knowledge of the intervention received. Regarding RoB for the studies that employed pre–post designs, the majority (*n* = 8) of the studies were rated fair quality (DiCarlo, 1988; McLean and Skinner, 1995; Silva et al., 1998; Bresnahan et al., 2019; Graham et al., 2019; Brizuela et al., 2020; Farkas et al., 2021); one was rated as good quality (Williams et al., 2020) and four were rated as poor quality (El-Sayed and Younesian, 2005; Jacobs, 2009; Harnish et al., 2017; Horiuchi and Okita, 2017). Details are summarized in Table 2 and in Supplementary Table 2. Due to insufficient quality and quantity in the included studies, performing a meta-analysis was deemed unsuitable.

### Effects of Arm-Crank Exercise on Cardiorespiratory Fitness

Fifteen studies reported outcomes of cardiorespiratory fitness using graded cardiopulmonary exercise testing, performed on an arm-crank ergometer to volitional exhaustion to identify peak oxygen consumption (V̇O_2peak_) and peak power output (Davis et al., 1987, 1991; DiCarlo, 1988; McLean and Skinner, 1995; El-Sayed and Younesian, 2005; Jacobs, 2009; Ordonez et al., 2013; Harnish et al., 2017; Horiuchi and Okita, 2017; Nightingale et al., 2017b; Bresnahan et al., 2019; Graham et al., 2019; Williams et al., 2020; Alrashidi et al., 2021; Farkas et al., 2021). Two studies used spirometry to assess pulmonary function and measured forced vital capacity (FVC) and maximum voluntary ventilation (MVV) (Silva et al., 1998; Brizuela et al., 2020). All studies using exercise testing reported a statistically significant (*P* < 0.05) increase in V̇O_2peak_ between pre- and post-intervention (effect size range, *g* = 0.19–5.14; large [≥0.8]: 6/15; medium [≥0.5]: 5/15; small [≥0.2]: 2/15; <0.2 or uncalculatable: 2/15). Of the two studies using spirometry, one reported no change in FVC and increased MVV after the intervention (Brizuela et al., 2020), whereas the other study reported increased FVC but no change in MVV between pre- and post-intervention values (Silva et al., 1998). Twelve studies reported the outcome of peak power output obtained from the graded cardiopulmonary exercise testing (DiCarlo, 1988; McLean and Skinner, 1995; El-Sayed and Younesian, 2005; Jacobs, 2009; Harnish et al., 2017; Horiuchi and Okita, 2017; Nightingale et al., 2017b; Bresnahan et al., 2019; Graham et al., 2019; Brizuela et al., 2020; Williams et al., 2020; Alrashidi et al., 2021). Of the twelve studies, ten reported a statistically significant increase in peak power after the ACE intervention (*g* = 0.05–5.91; large [≥0.8]: 3/10; medium [≥0.5]: 4/10; small [≥0.2]: 1/10; <0.2 or uncalculatable: 2/10) (DiCarlo, 1988; McLean and Skinner, 1995; El-Sayed and Younesian, 2005; Jacobs, 2009; Harnish et al., 2017; Horiuchi and Okita, 2017; Nightingale et al., 2017b; Bresnahan et al., 2019; Brizuela et al., 2020; Williams et al., 2020); two reported no change in peak power (Graham et al., 2019; Alrashidi et al., 2021). In accordance with the GRADE tool, the included studies reporting on cardiorespiratory fitness had an overall sample size of >100 participants and consistent results. Therefore, a moderate level of evidence is available to recommend ACE to improve cardiorespiratory fitness for adults with chronic SCI.

### Effects of Arm-Crank Exercise on Body Composition

Body composition was assessed in six studies using body weight, fat mass, lean body mass, body mass index, and waist circumferences as outcomes (Ordonez et al., 2013; Horiuchi and Okita, 2017; Nightingale et al., 2017b; Bresnahan et al., 2019; Graham et al., 2019; Farkas et al., 2021). Three studies (Bresnahan et al., 2019; Graham et al., 2019; Farkas et al., 2021) reported no differences in body composition outcomes between pre- and post-intervention. Two studies reported decreased body mass after the intervention (Horiuchi and Okita, 2017; Nightingale et al., 2017b), with trivial to small effect sizes (*g* = 0.03–0.26). Furthermore, two studies reported reduced waist circumferences values after the intervention (*g* = 0.32) (Ordonez et al., 2013; Horiuchi and Okita, 2017). Results were inconsistent from low quality studies, with a low number of participants (*n* = 55 in total). Hence, there is a very low level of evidence to support ACE altering body composition in adults with SCI.

### Effects of Arm-Crank Exercise on Cardiovascular Disease Risk Factors

There were nine included studies reporting outcomes relating to CVD risk factors (Davis et al., 1987; El-Sayed and Younesian, 2005; Harnish et al., 2017; Horiuchi and Okita, 2017; Nightingale et al., 2017b; Bresnahan et al., 2019; Graham et al., 2019; Alrashidi et al., 2021; Farkas et al., 2021).

#### Glucose Tolerance and Insulin Sensitivity

Five studies assessed fasting insulin (Harnish et al., 2017; Nightingale et al., 2017b; Bresnahan et al., 2019; Graham et al., 2019; Farkas et al., 2021) and 3 of those reported reduced values between pre- and post-intervention (*g* = 0.4–1.0) (Nightingale et al., 2017b; Bresnahan et al., 2019; Farkas et al., 2021). Two studies evaluated glycated hemoglobin and reported no change in values after the ACE (Horiuchi and Okita, 2017; Alrashidi et al., 2021). Four studies assessed homeostasis model assessment of insulin resistance (HOMA-IR) (Nightingale et al., 2017b; Bresnahan et al., 2019; Graham et al., 2019; Alrashidi et al., 2021); two reported a significant reduction in HOMA-IR (*g* = 0.53–1.09) (Nightingale et al., 2017b; Bresnahan et al., 2019), while the others reported a trend for a decrease (Graham et al., 2019; Alrashidi et al., 2021) after the ACE intervention. Furthermore, seven studies assessed fasting glucose but none reported differences between pre- and post-intervention (Harnish et al., 2017; Horiuchi and Okita, 2017; Nightingale et al., 2017b; Bresnahan et al., 2019; Graham et al., 2019; Alrashidi et al., 2021; Farkas et al., 2021).

#### Hyperlipidemia

There were seven studies assessing high-density lipoprotein cholesterol (HDL-C), low-density lipoprotein cholesterol (LDL-C), and triglycerides as the outcome measures of lipid profile (El-Sayed and Younesian, 2005; Horiuchi and Okita, 2017; Nightingale et al., 2017b; Bresnahan et al., 2019; Graham et al., 2019; Alrashidi et al., 2021; Farkas et al., 2021). The majority of the studies reported no change in HDL-C, LDL-C and triglycerides after the ACE intervention, compared with the baseline values (Horiuchi and Okita, 2017; Nightingale et al., 2017b; Bresnahan et al., 2019; Graham et al., 2019; Alrashidi et al., 2021; Farkas et al., 2021). There were two studies reporting decreased fasting triglycerides post intervention (El-Sayed and Younesian, 2005; Horiuchi and Okita, 2017) and 1 study reported increased HDL-C after the intervention (El-Sayed and Younesian, 2005). It was not possible to estimate effect sizes from these studies as mean values or standard deviation were not reported.

#### Hypertension

Five studies included outcomes relating to hypertension, including systolic blood pressure (SBP) and diastolic blood pressure (DBP) (Davis et al., 1987; Horiuchi and Okita, 2017; Nightingale et al., 2017b; Graham et al., 2019; Farkas et al., 2021). Of those, two reported decreased SBP after the ACE (*g* = 0.6–0.68) (Horiuchi and Okita, 2017; Farkas et al., 2021), while the other three studies reported no differences between pre- and post-intervention values (Davis et al., 1987; Nightingale et al., 2017b; Graham et al., 2019). No studies reported changes in DBP values post intervention.

#### Other Cardiovascular Disease Risk Factors

Some studies reported the effects of ACE on other CVD risk factors, such as inflammatory markers, adipokines, or vascular structure and function. Two studies assessed plasminogen activator inhibitor 1, with one study reporting a reduction (Horiuchi and Okita, 2017) and one reporting no change (Rosety-Rodriguez et al., 2014) in values after an ACE intervention. Two studies evaluated non-esterified fatty acids pre- and post-ACE; one study reported a decrease in non-esterified fatty acids after the ACE (Harnish et al., 2017), whereas the other study reported no change after the training (Nightingale et al., 2017b). Only one study (Rosety-Rodriguez et al., 2014) evaluated tumor necrosis factor-alpha, interleukin-6, leptin, and adiponectin in response to ACE training; concentrations of these markers decreased post ACE, except for adiponectin, which showed no difference after the training. Moreover, one study observed no change in arterial stiffness over 6 months of ACE (Alrashidi et al., 2021).

Overall, given half of the studies were rated with poor quality and results were inconsistent across the studies, there is a very low level of evidence to support the effectiveness of ACE in modifying CVD risk factors in adults with chronic SCI.

### Effects of Arm-Crank Exercise on Motor Function (Strength, Balance, and Mobility) and Health-Related Quality of Life

The effects of ACE on motor function and health-related QoL are rarely discussed. Of 17 included studies, three studies reported muscle strength (Jacobs, 2009; Horiuchi and Okita, 2017; Graham et al., 2019), two reported wheelchair mobility (DiCarlo, 1988; Bresnahan et al., 2019), one reported sitting balance (Williams et al., 2020), and one reported health-related QoL (Nightingale et al., 2018).

Of the three studies measuring muscle strength before and after the ACE (Jacobs, 2009; Horiuchi and Okita, 2017; Graham et al., 2019), only one study reported increased strength of the upper-body (Graham et al., 2019). Furthermore, two studies measuring wheelchair mobility reported an increase in wheelchair propulsion distance after the ACE intervention (DiCarlo, 1988; Bresnahan et al., 2019), albeit the effect sizes reported in the Bresnahan’s study (Bresnahan et al., 2019) and in the DiCarlo’s study (DiCarlo, 1988) were considerably different (0.3 and 7.6, respectively). In addition, one study assessing sitting balance reported inconclusive effects of ACE on sitting balance in individuals with SCI (Williams et al., 2020). Moreover, the effects of ACE on health-related QoL in individuals with SCI was assessed using the short-form 36 physical and mental component scores in one study (Nightingale et al., 2018), with increased health-related QoL after the ACE intervention.

Overall, the certainty of the body of evidence is very low and therefore it is unable to draw a recommendation to ACE influencing motor function or health-related QoL in adults with SCI. Certainty of evidence to individual outcome measures is summarized in Table 5.

**TABLE 5:** GRADES certainty of the evidence.

Outcomes	Direction of effect	Quality of evidence
Cardiorespiratory fitness	↑↑	⊕⊕⊕⃝
Body composition	↑?	⊕⃝⃝⃝
Cardiovascular disease risk factors	↑?	⊕⊕⊕⃝
Motor function (balance, strength, mobility)	↑?	⊕⃝⃝⃝
Health-related quality of life	↑?	⊕⊕⊕⃝

*↑↑ Strong for an intervention; ↑? Weak for an intervention; ⊕⊕⊕⃝ moderate quality of evidence; ⊕⃝⃝⃝very low quality of evidence.*

### Adverse Events

Five studies reported on exercise-related injuries (Silva et al., 1998; Dyson-Hudson et al., 2007; Ordonez et al., 2013; Nightingale et al., 2018; Williams et al., 2020). Two studies used the Wheelchair Users Shoulder Pain Index, which is a 15-item self-reported outcome measure that utilizes a visual analogue scale to interpret pain during transfers, wheelchair mobility, self-care, and general activities (Dyson-Hudson et al., 2007; Nightingale et al., 2018). Both studies reported no significant increases in the intensity of shoulder pain post-intervention. Another three studies explicitly reported that no participant reported exercise-related pain or injuries as a result of the intervention (Silva et al., 1998; Ordonez et al., 2013; Williams et al., 2020). Overall, evidence shows ACE to be a safe exercise modality for adults with chronic SCI.

## Discussion

This review shows consistent findings that ACE is effective in improving cardiorespiratory fitness in adults with chronic SCI. The review also shows that overall ACE is a safe exercise modality for adults with SCI. However, the review is unable to draw recommendations on ACE for altering body composition, CVD risk factors, motor function, or health-related QoL in adults with chronic SCI due to the low certainty of the body of evidence. This was compounded by the small sample sizes and inconsistent findings across studies. In addition, the majority of the studies included in this review are pre-post studies (76%), which inherently have a lower level of evidence. More high-quality, RCT studies are needed to draw conclusions on the effects of ACE on body composition, CVD risk factors, motor function, and health-related QoL. Identification and recruitment of disabled population groups represents a challenge (Cardenas and Yilmaz, 2006). Therefore, multi-center trials (within and across countries) may be necessary to overcome this to ensure adequately powered research studies and wider generalisability of the findings.

### Arm-Crank Exercise Improves Cardiorespiratory Fitness in Chronic Spinal Cord Injury

All included studies showed improvements in cardiorespiratory fitness after the ACE; the average improvement in V̇O_2peak_ was 21% (range: 6–94%), with a large variation across studies. This is in keeping with a previous systematic review in upper-body exercise training in adults with SCI reporting 17.6% ± 11.2% increases in V̇O_2peak_ (Valent et al., 2007). Although the group results suggest no relation between the magnitude of improvement in V̇O_2peak_ and the total number of exercise sessions (*r* = –0.14; *p* = 0.69), the study with the lowest number of training sessions (only 6 sessions) had the smallest increase in V̇O_2peak_ (only 6% higher than the baseline) (Harnish et al., 2017). The study using light-to-moderate-intensity ACE (i.e., Borg CR 10 scale between 2 and 3) also showed relatively small improvements (only 6% higher than the baseline) (Brizuela et al., 2020), compared with other studies using moderate-to-vigorous-intensity ACE. Indeed, Davis and colleagues (Davis et al., 1991) tested the effects of exercise volume (long vs. short) and intensity (vigorous vs. moderate) on cardiorespiratory fitness in paraplegic adults. Results showed that all groups improved cardiorespiratory fitness, except the group undertaking short duration and moderate intensity ACE. This indicates the effects of ACE on cardiorespiratory fitness can be achieved by prescribing an appropriate exercise volume and/or intensity. This might be relevant to people with higher level injuries who may be unable to achieve maximal exercise intensities due to impaired autonomic and motor functions.

Furthermore, the average improvement of peak power with ACE was 44%, ranging from 2 to 360%, considerably higher than the value (26.1% ± 16.5%) reported in the previous systematic review of upper-limb exercise training (Valent et al., 2007). The study with the highest number of exercise sessions (5 sessions/week, 16 weeks, 80 sessions in total) showed the greatest increase in peak power (3.6 times higher than the baseline value) (Farkas et al., 2021). However, its peak power at baseline was lower than studies with similar participant demographics (i.e., thoracic SCI only) (Jacobs, 2009; Ordonez et al., 2013; Harnish et al., 2017; Nightingale et al., 2017b), indicating participants either presented with a greater level of physical deconditioning or a regression to the mean artifact (Shephard, 2003). This highlights the importance of prescribing a sufficient volume and/or intensity of ACE in this population. Indeed, a recent cross-sectional study showed that participants with SCI performing higher habitual levels of physical activity had higher levels of cardiorespiratory fitness (Nightingale et al., 2019). The length of the ACE intervention ranged from 2 to 24 weeks and the most common frequency was three times per week; the average number of exercise sessions was 33 sessions. The majority of the studies used moderate-to-vigorous intensity ACE (i.e., >60% V̇O_2peak_ or >50% of heart rate reserve). Hence, when prescribing ACE to adults with SCI for improving cardiorespiratory fitness, exercise intensity and length of the intervention should be considered as important parameters

While the effect of ACE on cardiorespiratory fitness is consistent across the included studies and seemingly independent of injury level, small sample sizes and a large proportion of included studies having pre-post designs reduced the overall level of evidence. More studies are needed to increase the level of evidence from moderate to high.

### Arm-Crank Exercise on Body Composition and Cardiovascular Disease Risk Factors

The review is unable to conclude the effects of ACE on body composition or CVD risk factors due to inconsistent findings across the studies. It is well accepted that the effects of exercise on various health parameters are linked with the amount and intensity of exercise performed (Nightingale et al., 2019). For example, the World Health Organization (WHO) recommends at least 150–300 min/week of moderate-intensity aerobic activity (or 75–150 min/week of vigorous-intensity aerobic activity) for the general population to reduce CVD risk factors (Bull et al., 2020). These recommendations were drawn from evidence regarding the dose-response of physical activity and mortality in non-injured adults (Powell et al., 2011). Individuals with chronic SCI often have lower physical capacity (Dallmeijer et al., 1996), lower-limb skeletal muscle atrophy (Maggioni et al., 2003; Spungen et al., 2003) and impaired autonomic cardiovascular control (i.e. cervical and upper-thoracic SCI) (West et al., 2015), which may result in different responses to a period of exercise compared to non-injured adults. Amongst the included studies, the mean exercise time was ∼100 min per week (range: 90–200 min per week), with the majority of the studies employing moderate-to-vigorous intensity ACE. This exercise volume is below the lower end of WHO recommendations for the general population, however, above the SCI Action Canada recommendations for adults with SCI to engage 40 and 90 min of moderate-to-vigorous aerobic physical activity per week for fitness and for cardiometabolic health, respectively (Martin Ginis et al., 2018). Compared to lower-body exercise or whole-body exercise, there are fewer muscles activated during ACE and the muscles involved have a relatively smaller muscle mass, hence a reduced oxygen consumption and lower energy expenditure (Nightingale et al., 2017a). Consequently, ACE results in a smaller metabolic disturbance and generates less of an energy deficit to modulate body composition, compared with lower-body or whole-body exercise (McMillan et al., 2021). Furthermore, it has been reported that individuals with motor-complete tetraplegia utilize ∼50% less VO_2_ during exercise than individuals with motor-complete paraplegia (Holmlund et al., 2018). Consequently, Shea and colleagues (Shea et al., 2018) suggested that individuals with higher level injuries may require a greater exercise volume (>220 min per week) to overcome the unique low energy expenditure achieved in this population and observe a health benefit from upper-body exercise. Thus, future research is required to determine optimal ACE prescriptions to effectively and consistently modify body composition and CVD risk factors for adults with chronic SCI. This could be in conjunction with combined therapies such as circuit resistance training (Nash et al., 2001) or FES-evoked lower-limb exercise (Bekhet et al., 2021) to increase skeletal muscle mass (i.e., increasing basal metabolic rate). Given the restricted energy expenditure with arm-exercise, concurrently reducing energy intake through the introduction of a sustainable calorie deficit may more favorably modulate body composition. Currently, little is known with regards to whether a prescribed ACE intervention simply replaces other activity (termed substitution) or causes a compensatory increase in energy intake in response to a perceived state of deficit in this population (Nightingale et al., 2017b). These concepts may erode the effectiveness of upper-body exercise in modulating body composition and CVD risk (Nightingale and Bilzon, 2016), and additional studies accurately characterizing changes in habitual energy intake and expenditure are required.

Studies investigating other disease-specific CVD risk factors were included in this review. For example, plasminogen activator inhibitor 1 has a vital role in deep vein thrombosis, which is common after SCI (Chung et al., 2014). Additionally, arterial stiffness is a prognostic risk factor for future CVD events (Vlachopoulos et al., 2010) and is elevated in individuals with SCI (Phillips et al., 2012). Inflammatory markers are also elevated in the SCI population relative to able-bodied individuals (Davies et al., 2007) and chronic inflammation has been linked with CVD (Alfaddagh et al., 2020). However, these variables were reported in only one or two studies and results were unequivocal. Future research is needed to determine whether ACE may influence these non-traditional CVD risk factors in adults with SCI.

### Effects of Arm-Crank Exercise on Strength, Balance, Mobility, and Health-Related Quality of Life

Arm-crank exercise requires volitional movements of the upper-body and torso. However, only a few studies have reported the effects of ACE alone on strength of upper limbs, sitting balance, and wheelchair mobility in adults with chronic SCI.

In this review, two studies reported improved wheelchair mobility, assessed by distance of wheelchair propulsion achieved in 12 min after the ACE intervention (DiCarlo, 1988; Bresnahan et al., 2019). Improved cardiorespiratory fitness resulted in improved endurance, hence the increased distance of continuous wheelchair propulsion. Increased strength of upper limbs due to the ACE intervention could also contribute to the increase in wheelchair mobility, which is highly relevant to activities of daily living and ensuring independence. However, due to the low quality and quantity of studies reporting on these outcomes, further studies are needed to confirm the effect and optimal duration of an ACE intervention on strength and mobility.

The review is unable to draw a conclusion on the effects of the ACE on sitting balance in adults with SCI due to only one study reporting this outcome. Studies have reported involvement of the trunk during ACE (Mossberg et al., 1999; Dallmeijer et al., 2004) and trunk control is important in maintaining upright posture and stability, which is highly relevant to adults with SCI. More studies evaluating the influence of the ACE on postural control are required.

### Adverse Events

It should be noted that “no adverse affects” were reported in any of the papers reviewed, and in those which documented shoulder pain, no clinically meaningful change was noticed on a group level, suggesting arm-crank exercise does not increase shoulder pain. However, none of the papers documented any follow up assessments following the intervention, therefore, the long-term effects of ACE remain unknown.

### Limitations

A limitation of this review is that the majority of the studies included were pre-post designs, with only 4 RCTs. RCTs are regarded as the most rigorous study design to infer causality, however, there are challenges in carrying out such trials among the SCI population (Ginis and Hicks, 2005). Firstly, barriers to exercise such as transportation or financial issues can prevent motivated participants from adhering to interventions. This was observed in this review where some studies reported > 20% dropout rates (Bresnahan et al., 2019; Graham et al., 2019; Williams et al., 2020; Alrashidi et al., 2021). The complexity of individuals’ injuries results in a more frequent likelihood of secondary health complications (i.e. urinary tract infection, pressure sores, and over-use injuries), which could in part account for high dropout rates (Ginis et al., 2010).

Another limitation is that most papers do not differentiate the effects of ACE by level of injury. A recent systematic review questioned the effectiveness of aerobic exercise in cardiorespiratory fitness for individuals with high levels of SCI (i.e., tetraplegia) (Figoni et al., 2021). However, three studies in this review included only adults with cervical SCI (above T1) and showed a significant increase in aerobic fitness following an 8–10 weeks intervention, with percentage changes in V̇O_2peak_ ranging from 7 to 94% (DiCarlo, 1988; McLean and Skinner, 1995; Brizuela et al., 2020). Note that the two studies (McLean and Skinner, 1995; Brizuela et al., 2020) using light to moderate intensity based on the RPE or peak power output showed 7–8% increases in V̇O_2peak_, whereas the study (DiCarlo, 1988) using moderate-to-vigorous intensity based on heart rate reserved showed a 94% increase in V̇O_2peak._ This suggests that individuals with higher level injuries and impaired exercise-induced changes in cardiovascular control may still benefit from appropriate aerobic exercise prescription. In parallel, five studies recruited only adults with thoracic SCI and also reported increased cardiorespiratory fitness after the ACE intervention; the percentage changes at post-intervention also covered a wide range from 6 to 22% (Jacobs, 2009; Harnish et al., 2017; Horiuchi and Okita, 2017; Nightingale et al., 2017b; Farkas et al., 2021). This suggests that regardless of heterogeneity, both individuals with tetraplegia and paraplegia can benefit from the ACE intervention for cardiorespiratory fitness. A further consideration based on the neurological level of injury is that some studies used a percentage of participants’ heart rate reserve to prescribe the intensity of ACE. However, in participants with cervical and upper-thoracic SCI, sympathetic nervous system disruption results in cardiovascular blunting (whereby peak heart rate does not increase beyond 130 b.min^–1^). Therefore, there are concerns around prescribing exercise intensity using a percentage of peak heart rate responses in this population and the appropriateness of other strategies should be explored with future research. Additionally, the gender breakdown of 78% males reported across studies in this review reflects the ratio reported in the wider population (National Spinal Cord Injury Statistical Center, 2019). Finally, there were a few earlier research articles (year 1991 or before) that were excluded from the initial database search strategies, despite no limitation placed on the publication year; key papers were identified and one such paper was added to the review by hand (Davis et al., 1991).

## Conclusion

In summary, this systematic review provides evidence that ACE interventions benefit cardiorespiratory fitness in people living with chronic SCI. To achieve this effect, a sufficient volume and intensity of exercise is important. Based on the evidence included in this review, we suggest moderate-to-vigorous arm-crank exercise for 30–40 min per time, three times per week on a regular basis for promoting cardiorespiratory fitness in adults with chronic SCI. More high-quality RCTs with larger sample sizes assessing body composition, CVD risk factors, and motor function parameters are needed to inform and refine evidence-based arm-crank exercise guidelines for promoting health in this population.

## Data Availability Statement

The original contributions presented in the study are included in the article/Supplementary Material, further inquiries can be directed to the corresponding author.

## Author Contributions

EC, CL, TH, and TN performed the search of data bases and reference lists. SYC, EC, CL, and TH carried out the drafting of the manuscript. All authors contributed to the manuscript revisions and the approval of the final manuscript and performed quality assessment and data analysis.

## Conflict of Interest

The authors declare that the research was conducted in the absence of any commercial or financial relationships that could be construed as a potential conflict of interest.

## Publisher’s Note

All claims expressed in this article are solely those of the authors and do not necessarily represent those of their affiliated organizations, or those of the publisher, the editors and the reviewers. Any product that may be evaluated in this article, or claim that may be made by its manufacturer, is not guaranteed or endorsed by the publisher.
