# Supplementary material for: Effects of Arm-Crank Exercise on Fitness and Health in Adults With Chronic Spinal Cord Injury: A Systematic Review
Source: Front Physiol. 2022 Mar 17;13:831372. doi: 10.3389/fphys.2022.831372 (PMC8982085; doi:10.3389/fphys.2022.831372)
Supplement: Supplementary file 1 [file Table_1.DOCX]

**Supplementary Table 1. Search strategies.**

***PubMed***

| Search order | Search term | Search results |
| --- | --- | --- |
| 1 | "Spinal Cord Injuries"[Mesh] | 49763 |
| 2 | "Paraplegia"[Mesh] | 13121 |
| 3 | "Quadriplegia"[Mesh] | 8121 |
| 4 | arm ergometer | 722 |
| 5 | arm cycling | 7103 |
| 6 | arm crank | 732 |
| 7 | arm exercise | 11755 |
| 8 | arm training | 18170 |
| 9 | "Spinal Cord Injuries"[Mesh] AND arm ergometer | 73 |
| 10 | "Spinal Cord Injuries"[Mesh] AND arm cycling | 64 |
| 11 | "Spinal Cord Injuries"[Mesh] AND arm crank | 125 |
| 12 | "Spinal Cord Injuries"[Mesh] AND arm exercise | 332 |
| 13 | "Spinal Cord Injuries"[Mesh] AND arm training | 242 |
| 14 | 9 OR 10 OR 11 OR 12 OR 13 | 412 |
| 15 | "Paraplegia"[Mesh] AND arm ergometer | 47 |
| 16 | "Paraplegia"[Mesh] AND arm cycling | 27 |
| 17 | "Paraplegia"[Mesh] AND arm crank | 99 |
| 18 | "Paraplegia"[Mesh] AND arm exercise | 175 |
| 19 | "Paraplegia"[Mesh] AND arm training | 99 |
| 20 | 15 OR 16 OR 17 OR 18 OR 19 | 208 |
| 21 | "Quadriplegia"[Mesh] AND arm ergometer | 22 |
| 22 | "Quadriplegia"[Mesh] AND arm cycling | 20 |
| 23 | "Quadriplegia"[Mesh] AND arm crank | 33 |
| 24 | "Quadriplegia"[Mesh] AND arm exercise | 94 |
| 25 | "Quadriplegia"[Mesh] AND arm training | 77 |
| 26 | 21 OR 22 OR 23 OR 24 OR 25 | 133 |
