# Supplementary material for: Effects of Arm-Crank Exercise on Fitness and Health in Adults With Chronic Spinal Cord Injury: A Systematic Review
Source: Front Physiol. 2022 Mar 17;13:831372. doi: 10.3389/fphys.2022.831372 (PMC8982085; doi:10.3389/fphys.2022.831372)
Supplement: Supplementary file 2 [file Table_2.docx]

**Supplementary Table 2. Risk of Bias assessments.**

| Cochrane RoB 2.0 for randomised controlled trial | | | | | | | | | | | | | | | | | | | | | |
| --- | --- | --- | --- | --- | --- | --- | --- | --- | --- | --- | --- | --- | --- | --- | --- | --- | --- | --- | --- | --- | --- |
| Criteria  Study | 1.Randomisation | | 2.Interventions | | | | | | 3.Missing outcome data | | | 4.Outcome Measurement | | 5.Reported results | | | | | Overall | | |
| Davis et al. 1991 [54] | High | | Some concerns | | | | | | High | | | Low | | Some concerns | | | | | High | | |
| Dyson-Hudson et al. 2007 [48] | High | | Low | | | | | | Low | | | High | | Low | | | | | High | | |
| Ordonez et al. 2013 [38]  Rosety-Rodriguez et al. 2014 [39] | Low | | Low | | | | | | Low | | | Low | | Low | | | | | Low | | |
| Nightingale et al. 2017 [40]  Nightingale et al. 2018 [23]  RCT | Low | | Low | | | | | | Low | | | Low | | Low | | | | | Low | | |
| Cochrane ROBINS-I for non-randomised controlled trial | | | | | | | | | | | | | | | | | | | | | |
| Criteria  Study | 1.Confounding | | 2.Selection of participants | | | 3.Classification of interventions | | | 4.Deviations from intended interventions | | | 5.Missing outcome data | | 6.Outcome measurement | | | 7.Selective reporting | | | Overall | |
| Davis et al. 1987 [50] | Moderate | | Low | | | Moderate | | | No information | | | Serious | | Moderate | | | Low | | | Serious | |
| NIH quality assessment tool for before-after (Pre-Post) study with no control group | | | | | | | | | | | | | | | | | | | | | |
|  | Q1 | Q2 | | Q3 | Q4 | | Q5 | Q6 | | Q7 | Q8 | | Q9 | | Q10 | Q11 | | Q12 | | | Overall |
| Dicarlo, 1988 [21] | Y | Y | | N | NR | | N | Y | | Y | N | | NR | | Y | N | | NA | | | Fair |
| McLean et al. 1995 [49] | Y | Y | | Y | N | | N | N | | Y | N | | Y | | Y | N | | NA | | | Fair |
| Silva et al. 1998 [53] | Y | Y | | Y | NR | | N | Y | | Y | N | | NR | | Y | N | | NA | | | Fair |
| El-Sayed et al. 2005 [43] | Y | N | | NR | NR | | N | Y | | Y | N | | NR | | Y | N | | NA | | | Poor |
| Jacobs 2009 [42] | Y | N | | N | NR | | N | Y | | Y | NR | | NR | | Y | N | | NA | | | Poor |
| Harnish et al. 2017 [52] | Y | Y | | N | CD | | N | Y | | Y | N | | N | | N | Y | | NA | | | Poor |
| Horiuchi et al. 2017 [44] | Y | CD | | N | CD | | N | Y | | Y | N | | NR | | Y | N | | NA | | | Poor |
| Bresnahan et al. 2019 [24] | Y | Y | | Y | NR | | N | Y | | Y | N | | N | | Y | N | | NA | | | Fair |
| Graham et al. 2019 [51] | Y | Y | | Y | NR | | N | Y | | Y | N | | N | | Y | N | | NA | | | Fair |
| Brizuela et al. 2020 [47] | Y | Y | | N | NR | | N | Y | | Y | N | | NR | | Y | N | | NA | | | Fair |
| Williams et al. 2020 [25] | Y | Y | | Y | CD | | N | Y | | Y | N | | Y | | Y | N | | NA | | | Good |
| Alrashidi et al. 2021 [41] | Y | Y | | Y | N | | N | Y | | Y | N | | N | | Y | N | | NA | | | Fair |
| Farkas et al. 2021 [22] | Y | Y | | N | NR | | N | Y | | Y | Y | | NR | | Y | N | | NA | | | Fair |
